# Transcriptional Regulation of the Meiotic Cohesin Subunit RAD21L by Methylation of Two CpG Islands in Its Promoter and Characterization of Its Interactions with Histones

**DOI:** 10.3390/cells15201859

**Published:** 2026-10-13

**Authors:** Wuyun Dalai, Keiichi Saeki, Wuen Jiya, Xin Jin, Mei Rong

**Affiliations:** 1 College of Life Science and Technology, Inner Mongolia Normal University, Hohhot 010022, China; wuyundalai@imnu.edu.cn (W.D.);; 2 Key Laboratory of Biodiversity Conservation and Sustainable Utilization in Mongolian Plateau for College and University of Inner Mongolia Autonomous Region, Hohhot 010022, China; 3 Laboratory of Microbiology and Immunology, Graduate School of Agricultural Science, Kobe University, 1-1 Rokkodai-cho, Nada-ku, Kobe 657-8501, Japan

**Keywords:** cohesin, RAD21L, DNA methylation, transcriptional regulation, histone modifications, chromatin anchoring

## Abstract

**Highlights:**

**What are the main findings?**

- We identified, for the first time, a two-CpG-island model of transcriptional regulation for the meiosis-specific cohesin gene *Rad21l*. We constructed a single-base-resolution methylation map covering 56 CpG sites, and in vitro methylation experiments directly confirmed that DNA methylation represses promoter activity, thereby elucidating how reproductive-tissue-specific hypomethylation regulates gene expression.
- We found, for the first time, that the encoded RAD21L protein interacts with histones in a modification-state-specific manner: interactions were detected with monomethylated, phosphorylated, and acetylated histones, but not with trimethylated histones. Integrated multi-omics analyses identified KLF4 as a key transcription factor, leading us to propose a novel coordinated dual epigenetic regulatory mechanism involving DNA methylation at the two CpG islands and histone modifications.

**What are the implications of the main findings?**

- This epigenetic regulatory mechanism provides insight into the molecular basis of normal meiotic progression and further refines our understanding of the epigenetic regulatory network governing germ cell development.
- This mechanism provides an important theoretical basis for understanding how abnormalities in *Rad21l* lead to reproductive disorders, including meiotic defects, sperm abnormalities, and infertility, and for identifying potential diagnostic and therapeutic targets for these disorders.

**Abstract:**

The meiosis-specific cohesin α-kleisin subunits REC8 and RAD21L regulate higher-order chromatin organization and germline-specific transcription in male germ cells. *Rad21l* exhibits high, testis-specific expression in mice, but the epigenetic mechanisms governing its promoter activity and those underlying its chromatin targeting and anchoring remain unclear. To address these questions, we investigated *Rad21l* using dual-luciferase reporter assays, in vitro methylation, bisulfite sequencing, molecular docking, transcriptome sequencing, and co-immunoprecipitation. We identified a distal regulatory region and mapped the core promoter of *Rad21l* to the −228 to −3 bp region. We further demonstrated that methylation of the promoter CpG islands represses its transcription. Molecular docking simulations suggested that KLF4 could bind a promoter sequence containing CpG5 and that methylation would not disrupt this interaction. Methylation was hypothesized to induce conformational remodeling of KLF4, shift its ability to recruit cofactors, and thereby enable bidirectional transcriptional regulation. Co-immunoprecipitation confirmed the co-occurrence of α-kleisin proteins with the meiosis-specific histone H1t and histone H3, detected modified H3 forms including H3K9me1 and H3S10phos, and showed their association with HP1β. Histone modifications may regulate interaction affinity through long-range allosteric effects. This study proposes a working model for tissue-specific transcriptional regulation of *Rad21l* involving CpG methylation and KLF4 and establishes a hypothetical model of histone-dependent RAD21L chromatin anchoring, providing a basis for investigating male meiosis and identifying infertility-related targets.

## 1. Introduction

Meiosis is fundamental to male reproductive development in mammals. It comprises one round of DNA replication followed by two successive nuclear divisions, during which homologous chromosomes pair, synapse, recombine, and segregate accurately, thereby ensuring correct gamete ploidy and generating genetic diversity [1]. Disruption at any stage of meiosis can lead to defective synapsis, impaired recombination, or meiotic arrest, ultimately resulting in meiosis arrest or abnormal spermatogenesis. These meiotic defects are important molecular contributors to idiopathic male infertility [2,3]. The cohesin complex maintains meiotic chromosome architecture, mediates higher-order chromatin remodeling, and regulates germline-specific transcriptional programs, with distinct functions during mitosis and meiosis [4,5]. The functional specificity of cohesin is determined primarily by its core α-kleisin subunit. Members of this subfamily regulate chromosomal anchoring, cohesion, and three-dimensional genome architecture through specific protein interactions and spatiotemporal expression patterns [6]. Mammalian germ cells express two meiosis-specific α-kleisin subunits, REC8 and RAD21L. Although they differ substantially in spatiotemporal expression, chromatin localization, and biological function, REC8 and RAD21L act cooperatively to ensure orderly meiotic progression [7,8,9].

REC8 is the best-characterized meiotic cohesin subunit. It is enriched along chromosome arms and at centromeres, where it supports stable sister chromatid cohesion, synaptonemal complex assembly, DNA double-strand break repair, and homologous recombination; loss of REC8 results in complete meiotic arrest [10]. Recent studies have shown that germline-specific *Rec8* expression is epigenetically regulated by promoter CpG methylation. Methylation of the core promoter is inversely correlated with transcriptional activity, making REC8 a useful model for investigating the epigenetic regulation of α-kleisin family genes [11]. RAD21L is another key α-kleisin subunit in male meiosis and shows strict tissue specificity. It is highly expressed in mouse germ cells, whereas its expression is minimal or absent in somatic cells [12]. Recent studies have further demonstrated that, rather than primarily mediating canonical sister chromatid cohesion, RAD21L functions as a male germ cell-specific regulator of three-dimensional genome organization. It remodels chromatin topology during meiotic prophase, facilitates the formation of chromatin loop domains, and activates germline-specific transcriptional programs, thereby playing a central role in maintaining spermatogenic homeostasis and male fertility [1]. Although the cytological functions of RAD21L are becoming clearer, two key questions remain unresolved: the epigenetic mechanisms governing its tissue-specific transcription and the structural basis for its precise anchoring to meiotic chromatin.

Spatiotemporally regulated gene expression is fundamental to cell differentiation and tissue specialization. Promoter CpG island methylation is a conserved mechanism of epigenetic regulation in eukaryotes. It modulates transcription by altering promoter chromatin accessibility, impeding transcription factor binding, or recruiting repressive complexes [13,14]. Both activation and silencing of germline-specific genes depend strongly on tissue-specific promoter methylation patterns, whereas aberrant methylation can disrupt reproductive development [15,16]. Studies of the related cohesin subunit REC8 have shown that differential CpG methylation within its core promoter is central to maintaining germline-specific expression, suggesting that α-kleisin family genes may share a conserved methylation-based regulatory mechanism [11]. However, the location of the *Rad21l* core promoter, the effect of methylation on its activity, and the roles of key transcription factors in this regulation remain unclear. Krüppel-like factor 4 (KLF4) plays important roles in germ cell fate determination and epigenetic control of transcription. KLF4 has been proposed to respond to DNA methylation through conformational remodeling, potentially shifting between transcriptional activation and repression; it is also involved in regulating genes required for reproductive development [17,18]. However, in vivo experimental evidence is still lacking to establish whether KLF4 specifically binds the *Rad21l* promoter and mediates methylation-dependent transcriptional regulation.

Precise anchoring of proteins to chromatin is a prerequisite for their structural and transcriptional regulatory functions. The specialized microenvironment of meiotic chromatin is shaped by germline-specific histone variants and histone post-translational modifications [4,19]. H1 histone family, member T (H1t), is a testis-specific linker histone variant expressed predominantly in pachytene spermatocytes. Compared with somatic H1 subtypes, H1t binds nucleosomes more weakly, promoting a more open meiotic chromatin state that may facilitate the chromatin anchoring of cohesins and recombination and repair factors [20,21]. Methylation, phosphorylation, and acetylation of the histone H3 N-terminal tail are key modifications that modulate the affinity and specificity of protein-histone interactions. By altering local charge distribution and peptide-chain flexibility, these modifications can influence the localization of chromatin-binding proteins through allosteric effects [22,23]. In addition, HP1 family proteins are central regulators of higher-order chromatin organization. By recognizing histone modifications, they contribute to the maintenance of euchromatin-heterochromatin boundaries and participate broadly in meiotic chromatin remodeling [24,25]. Previous studies indicate that RAD21L participates in remodeling the three-dimensional organization of meiotic chromatin. However, direct evidence that RAD21L binds H1t, post-translationally modified H3, or HP1 proteins is lacking, and the interaction modes, structural determinants, and functional significance of these associations remain unknown. These gaps limit our mechanistic understanding of RAD21L localization to chromatin.

To address these gaps, we investigated mouse *Rad21l* from two complementary perspectives: epigenetic regulation of its transcription and localization of the encoded protein to chromatin. First, we used quantitative real-time PCR (RT-qPCR) to confirm the tissue-specific expression pattern of *Rad21l*. We then combined dual-luciferase reporter assays using promoter deletion constructs with in vitro methylation to map the core promoter of *Rad21l* and determine how CpG methylation affects promoter activity. We also used bioinformatics and molecular docking to assess the potential role of KLF4 in methylation-dependent transcriptional regulation of *Rad21l*. In parallel, we used co-immunoprecipitation (co-IP) to examine interactions of α-kleisin family proteins with the testis-specific linker histone H1t, modified forms of H3, and HP1 chromatin regulators. We then used molecular docking to predict the spatial organization and hydrogen-bonding patterns of these complexes and to explore how RAD21L may recognize the meiotic chromatin microenvironment and achieve targeted chromatin anchoring.

This study systematically elucidated a novel mechanism of tissue-specific *Rad21l* transcriptional regulation mediated by CpG methylation and developed a molecular model in which RAD21L is targeted to meiotic chromatin through specific interactions with histones. It fills a gap in research on the upstream and downstream regulatory networks of *Rad21l* and provides important experimental evidence and theoretical support for further elucidating chromatin remodeling during mammalian meiosis, understanding the molecular regulatory networks of male reproductive development, and exploring infertility-associated pathogenic targets.

## 2. Materials and Methods

### 2.1. Materials

All testicular, ovarian, and liver tissues used in the experiments were obtained from ICR mice purchased from Beijing Vital River Laboratory Animal Technology Co., Ltd. (Beijing, China).

### 2.2. RNA Extraction and Quantitative Real-Time PCR (RT-qPCR)

Total RNA was extracted from tissue samples using RNAiso Plus reagent (Takara, Kusatsu, Japan). After homogenization and lysis at room temperature, the samples were centrifuged at 12,000× *g* at 4 °C, and the supernatants were collected. Total RNA was purified by chloroform extraction, isopropanol precipitation, and washing with 75% ethanol. The RNA pellet was air-dried at room temperature and dissolved in DEPC-treated water.

Reverse transcription was performed in two steps using the PrimeScript RT Reagent Kit with gDNA Eraser (Takara) according to the manufacturer’s instructions. All reaction mixtures were prepared on ice. The gDNA elimination step was performed first by incubating the reaction at 42 °C for 2 min to remove genomic DNA. A 20 μL reverse-transcription reaction mixture was then prepared, incubated at 37 °C for 15 min for cDNA synthesis, and heated at 85 °C for 5 s to terminate the reaction. The cDNA products were stored at 4 °C for short-term use or at −20 °C for long-term storage.

Quantitative PCR was performed in 20 μL reaction mixtures using SYBR Premix Ex Taq (Takara) on an Applied Biosystems 7500 Real-Time PCR System (Thermo Fisher Scientific, Waltham, MA, USA). β-actin served as the reference gene for normalization of mouse *Rad21l* mRNA expression. The primer sequences were as follows: *Rad21l*, forward 5′-AGAAGTGGGAAACCAGCAGA-3′ and reverse 5′-ACCCACAGATTCATCAGTCACA-3′; β-actin, forward 5′-GGCTGTATTCCCCTCCATCG-3′ and reverse 5′-CCAGTTGGTAACAATGCCATGT-3′. The PCR program consisted of an initial denaturation at 95 °C for 30 s, followed by 40 cycles of denaturation at 95 °C for 5 s and annealing/extension at 60 °C for 34 s. Amplification specificity was verified by melting-curve analysis and agarose gel electrophoresis. Relative *Rad21l* expression across tissues was calculated using the 2^−ΔΔCt^ method.

### 2.3. Dual-Luciferase Reporter Assay

A 3000-bp segment of the mouse *Rad21l* gene (GenBank accession no. JN955050.1), encompassing an intron, exon I, and the upstream regulatory region, was selected for transcriptional regulatory analysis. Twelve primer pairs (Table 1) were designed to amplify DNA fragments for generating serial 5′-deletion constructs of the *Rad21l* promoter.

Genomic DNA was extracted from testis tissue using the MiniBEST Universal Genomic DNA Extraction Kit (Takara) and used as the template for high-fidelity PCR amplification with KOD-Plus-Neo (Toyobo, Osaka, Japan). PCR was performed on a Biometra Tone 96G (Jena, Germany) gradient thermal cycler in a total volume of 50 μL containing 1 U KOD-Plus-Neo DNA polymerase, 5 μL 10 × KOD-Plus-Neo PCR buffer, 0.2 mM dNTPs, 0.2 μM each of the forward and reverse primers, 1.5 mM MgSO_4_, and 100 ng template DNA. The cycling program consisted of an initial denaturation at 94 °C for 2 min, followed by 30 cycles of denaturation at 98 °C for 10 s and extension at 68 °C for 30 s. After separation by 1% agarose gel electrophoresis, the PCR products were purified using a SanPrep Column DNA Gel Extraction Kit (Sangon Biotech, Shanghai, China) and stored at −20 °C until use.

The target DNA fragments were inserted into the pMD20-T vector (Takara) by TA cloning. After sequence verification, the inserts were excised by double digestion with *Nhe*I and *Hind*III and subcloned into the corresponding restriction sites of the pGL3-Basic luciferase reporter vector (Promega, Madison, WI, USA) to generate the recombinant luciferase reporter constructs.

293T cells were seeded in 24-well plates at 30–40% confluence, with three biological replicates per group. Plasmid transfection was performed 24 h after seeding. For each well, 450 ng of the target construct and 50 ng of the pRL-TK internal control plasmid (500 ng total DNA) were mixed with 1.5 μL of transfection reagent. The transfection mixture was added dropwise to the corresponding wells and distributed evenly.

At 48 h post-transfection, dual-luciferase activity was measured using the Dual-Luciferase Reporter Assay System (Promega, E1910) according to the manufacturer’s instructions. The culture medium was removed, the cells were gently washed once with 1 × PBS, and all residual liquid was aspirated. Cells in each well were lysed with 200 μL of 1 × Passive Lysis Buffer (PLB) equilibrated to room temperature. The plate was gently agitated at room temperature for 15 min, and the entire cell lysate was collected. Luminescence measurements were performed in opaque white 96-well plates, and samples were protected from light throughout the procedure. For each measurement, 10 μL of cell lysate was mixed with 50 μL of Luciferase Assay Reagent II (LAR II) and incubated for 2 s before firefly luciferase activity (LUC) was measured. Immediately thereafter, 50 μL of Stop & Glo reagent was added and mixed, and the sample was incubated for 2 s before Renilla luciferase activity (REN) was measured. Renilla luciferase activity was used to normalize for transfection efficiency, and relative luciferase activity (LUC/REN) was calculated for each group.

### 2.4. In Vitro Methylation

For each of the 12 luciferase reporter constructs, a 20 μL reaction was prepared with 900 ng plasmid DNA, 2 μL of 10 × M.SssI buffer, 0.4 μL of 50 × S-adenosylmethionine (SAM), and 1 μL of CpG methyltransferase (M.SssI, Thermo Scientific, Waltham, MA, USA). After incubation at 37 °C for 15 min, the enzyme was inactivated by heating at 65 °C for 20 min. The reaction products were purified using a PCR purification kit, and promoter activity was measured using the dual-luciferase reporter assay system. Each experiment was performed independently at least three times.

### 2.5. DNA Extraction and Bisulfite Sequencing PCR (BSP)

Genomic DNA was extracted from each tissue sample using the MiniBEST Universal Genomic DNA Extraction Kit (Takara) according to the manufacturer’s instructions. After quantification, genomic DNA was stored at −20 °C until use. 1 μg of genomic DNA was subjected to bisulfite conversion using the EpiTect Fast DNA Bisulfite Kit (Qiagen, Venlo, The Netherlands), and the converted DNA was used as the template for PCR amplification. Each 50 μL PCR mixture contained 1.25 U EpiTaq HS polymerase (Takara), 5 μL 10× EpiTaq PCR Buffer, 2.5 mM MgCl_2_, 1.2 mM dNTP mixture, 0.4 μM each of the forward and reverse primers, and 50 ng template DNA. The primers used to amplify the target region of bisulfite-converted *Rad21l* are listed in Table 2. PCR was performed using 40 cycles of denaturation at 98 °C for 10 s, annealing at 55–60 °C for 30 s, and extension at 72 °C for 60 s. The PCR products were separated by 1% agarose gel electrophoresis and purified using a SanPrep Column DNA Gel Extraction Kit (Sangon Biotech, Shanghai, China). The purified PCR products were cloned into the pMD20-T vector (Takara). Following blue–white screening, plasmid DNA was extracted from white colonies; positive clones were identified by rapid restriction digestion and subsequently sequenced by Sangon Biotech Co., Ltd. (Shanghai, China).

For the BSP analysis, three biological samples were included for each tissue, and 10 independent clones from each sample were selected for sequencing. Thus, 30 clone sequences were obtained per tissue for descriptive methylation analysis. The clones were subsamples; the number of biological replicates was three (*n* = 3).

### 2.6. Bioinformatics Analysis

CpG island prediction: CpG islands in the mouse *Rad21l* promoter region were predicted using the online MethPrimer tool (http://www.urogene.org/, accessed on 8 January 2026).

Transcription factor-binding site prediction: Transcription factor-binding sites within the CpG sequences were predicted using the JASPAR database (http://jaspar.genereg.net/, accessed on 15 January 2026). The relative profile score threshold was set to 95%, and the JASPAR CORE non-redundant matrix collection was selected.

KLF4-DNA molecular docking: AlphaFold 3 was used to predict the structures of complexes between the KLF4 transcription factor and DNA. The predicted protein and DNA structures were submitted to the HDOCK server. A global search and scoring procedure was applied to KLF4 and DNA fragments representing two methylation states, and the complex conformation with the most favorable energy was selected. PyMOL (The PyMOL Molecular Graphics System, Version 2.6) was used to visualize the selected complexes in three dimensions and analyze their interactions.

Protein–protein molecular docking: Three-dimensional structures of the target proteins were downloaded from the UniProt database. Protein–protein docking was performed using HADDOCK, and the resulting complexes were analyzed using PRODIGY to identify interacting amino acid residues and calculate binding energies. PyMOL was used to visualize the docking results and generate three-dimensional representations of the complexes.

### 2.7. RNA-Seq and Data Analysis

Testis, liver, and ovary samples dissected from mice were immediately transferred to RNase-free tubes, snap-frozen in liquid nitrogen, and sent to Shanghai Majorbio Bio-pharm Technology Co., Ltd. (Shanghai, China) for transcriptome analysis. Each group included three biological replicates. Total RNA was extracted using TRIzol reagent, and RNA integrity and concentration were assessed using an Agilent 5300 Bioanalyzer (Agilent Technologies, Inc., Santa Clara, CA, USA) and a NanoDrop 2000. Strand-specific mRNA libraries were prepared using the Illumina Stranded mRNA Prep, Ligation kit (Ilumina, San Diego, CA, USA), and 2 × 150 bp paired-end sequencing was performed on the NovaSeq X Plus/DNBSEQ-T7 sequencing platform. All transcriptomic bioinformatics analyses were conducted on the Majorbio Cloud Platform (https://cloud.majorbio.com, accessed on 25 February 2026). Raw sequencing reads were filtered using fastp to remove adapters, low-quality sequences, and sequences containing ambiguous bases. The resulting clean reads were aligned to the reference genome using HISAT2. Gene expression was quantified using RSEM, and TPM (transcripts per million reads) was used to normalize expression levels. Differentially expressed genes were identified from the read count matrix using DESeq2, with selection criteria of |log_2_FC| ≥ 1 and FDR < 0.05; the Benjamini–Hochberg (BH) method was used for multiple-testing correction. Goatools (version 1.2.4) and the Python SciPy package (version 1.10.1) were used for Gene Ontology (GO) and Kyoto Encyclopedia of Genes and Genomes (KEGG) (Kanehisa Laboratories, Kyoto, Japan) functional enrichment analyses, respectively. Terms with a corrected FDR < 0.05 were considered significantly enriched. To explore interactions among the target genes, the STRING database (https://string-db.org, accessed on 3 March 2026) was used to construct a protein–protein interaction (PPI) network. The PPI network was visualized using Cytoscape (version 3.9.1), and the Cytoscape plugin MCODE (version 2.0.0) was used to identify key functional gene modules.

### 2.8. Co-Immunoprecipitation (co-IP) and Immunoblotting

For co-IP, testis extracts were incubated with an antibody against one of three cohesin subunits: RAD21, RAD21L, or REC8 [26] for 1.5 h at 4 °C with rotation. Protein A-sepharose beads (GE Healthcare, Uppsala, Sweden) was then added, and incubation was continued for 1 h. The beads were washed five times with TNE buffer (20 mM Tris-HCl, pH 7.4, 150 mM NaCl, 2 mM EDTA, 1% Nonidet P-40, 50 mM NaF, 1 mM Na_3_VO_4_, and 5 mM 2-mercaptoethanol), and the immunoprecipitated proteins were eluted by boiling for 3 min in SDS sample buffer containing 62.5 mM Tris-HCl (pH 6.8), 1% SDS, 10% glycerol, 50 mM dithiothreitol, and 0.02% bromophenol blue. The eluates were separated by SDS-PAGE on 12.5% polyacrylamide gels and transferred to polyvinylidene fluoride (PVDF) membranes (Thermo Scientific, Billerica, MA, USA) for immunoblotting. A rabbit polyclonal antiserum against H1t was prepared in our laboratory, and its suitability for immunoblotting was validated in our previous experiments. The remaining primary antibodies were as follows: anti-H3 (ab1791, Abcam), anti-H1 C-terminal (MA5-13750, Thermo Fisher Scientific, Waltham, MA, USA), anti-H3K4me1 (9723, Cell Signaling Technology, Danvers, MA, USA), anti-H3K4me3 (07-473, MilliporeSigma, Burlington, MA, USA), anti-H3K9me1 (ab9045, Abcam, Cambridge, UK), anti-H3K9me3 (07-442, MilliporeSigma), anti-H3K27me3 (61018, Active Motif, Carlsbad, CA, USA), anti-H3S10phos (06-570, MilliporeSigma), anti-H3S28phos (07-145, MilliporeSigma), anti-H3K14acetyl (06-911, MilliporeSigma), anti-HP1β (ab10811, Abcam), and anti-HP1γ (H2034-52, Bethyl Laboratories, Montgomery, TX, USA). The membranes were probed with these primary antibodies. Bound primary antibodies were detected using horseradish peroxidase (HRP)-conjugated secondary antibodies (GE Healthcare), and signals were developed using ECL Prime Western Blotting Detection Reagent (GE Healthcare).

### 2.9. Statistical Analysis

IBM SPSS Statistics (version 27.0) was used to analyze correlations between *Rad21l* expression levels and the methylation status of 56 CpG sites across tissues. Two-tailed Pearson correlation analysis was used to assess the linear relationship between relative gene expression and DNA methylation levels. *p* < 0.05 was considered statistically significant.

## 3. Results

### 3.1. Activity Analysis of the Proximal Core Promoter and Distal Upstream Regulatory Regions of the Rad21l Gene

**Figure 1:**
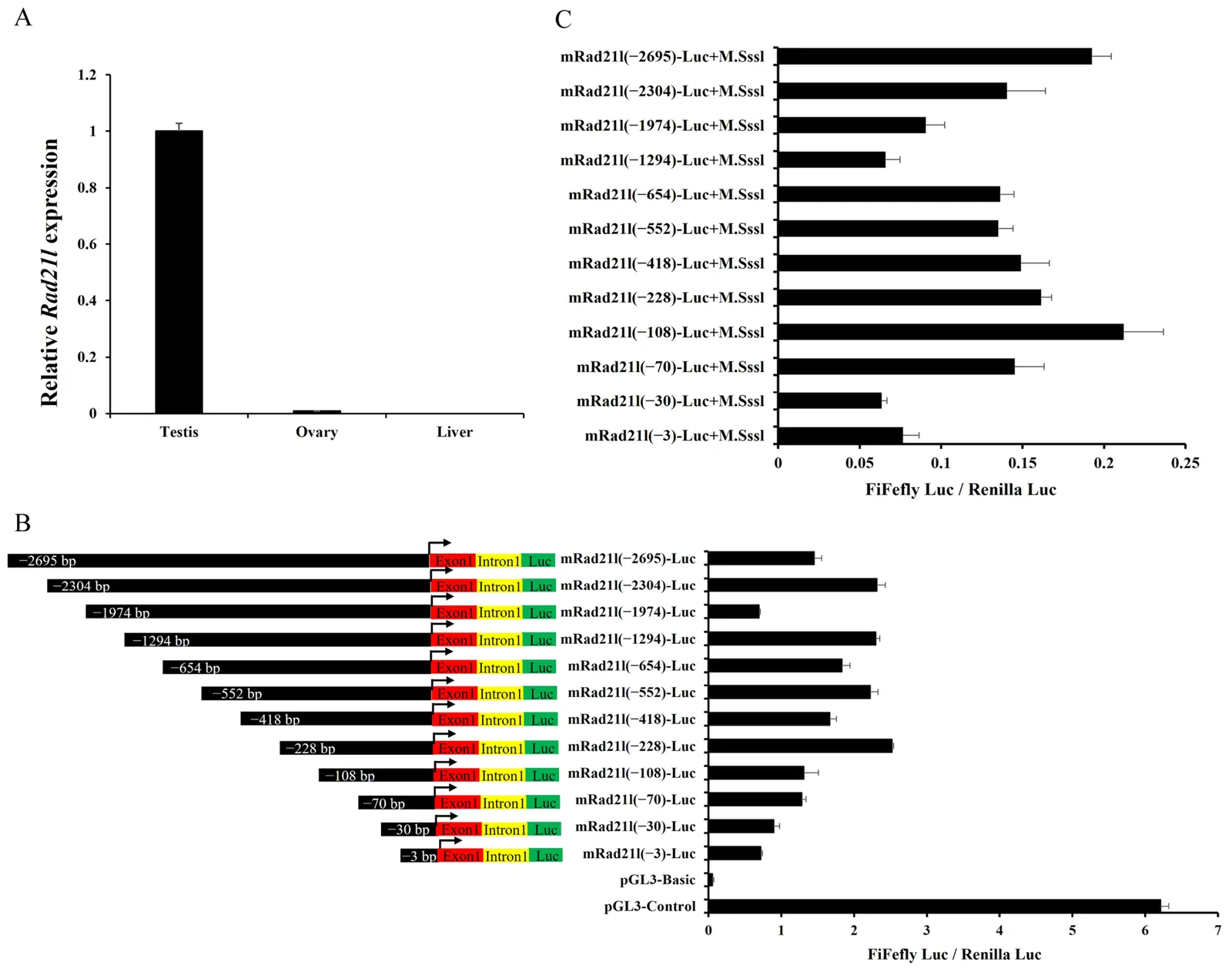
Tissue-specific expression pattern of *Rad21l* and identification of functional regions in its promoter. (**A**) *Rad21l* expression levels in mouse testis, ovary, and liver. Data are presented as the mean ± SD of three independent biological replicates. (**B**) Dual-luciferase reporter assay of serially 5′-deleted *Rad21l* promoter constructs. Schematics of promoter constructs of different lengths are shown on the left, and the relative luciferase activities of the 12 constructs after transfection are shown on the right. (**C**) Effects of in vitro methylation on the promoter activities of the serial 5′-deletion constructs. Bars represent the mean ± SD of three independent experiments.

Previous studies have shown that the mouse *Rad21l* gene exhibits a pronounced tissue-specific expression pattern [26]. In this study, RT-qPCR was used to further validate and quantify its transcript levels across tissues. *Rad21l* transcripts were undetectable in the liver, whereas transcript abundance in the testis was approximately 122-fold higher than that in the ovary (Figure 1A), suggesting that its expression is tightly regulated at the transcriptional level.

To investigate the molecular basis of transcriptional regulation of the *Rad21l* gene, we selected a regulatory region comprising an intron, exon I, and 2695 bp of upstream sequence (total length, 3000 bp) and generated 12 serially truncated dual-luciferase reporter constructs. Reporter activity was then measured to map the mouse core promoter and identify key cis-regulatory elements. pGL3-Basic served as the negative control (LUC/REN = 0.050), and pGL3-Control served as the positive control (LUC/REN = 6.210). All 12 serially truncated reporter constructs exhibited promoter activity; however, the transcriptional activity of each truncated fragment was significantly lower than that of the positive control, pGL3-Control (Figure 1B).

Comparison of luciferase activity among the serially truncated fragments identified three key regulatory regions. First, deletion of the distal −2304 to −1974 bp region reduced the LUC/REN ratio from 2.314 to 0.692, representing a 3.3-fold decrease in activity. Second, further deletion of the distal −1974 to −1294 bp region increased the LUC/REN ratio from 0.692 to 2.296, significantly restoring promoter activity. Third, truncation of the proximal −228 to −3 bp region significantly reduced the LUC/REN ratio from its peak value of 2.520 to 0.715, representing a 3.52-fold decrease and the largest reduction among all fragments. Notably, this region lacks features typical of housekeeping-gene promoters. Although it lacks a TATA-box element, it contains flexible AT-rich DNA sequences. Such sequences readily undergo bending and wrapping and can directly recruit TATA-box-binding protein (TBP), a conserved basal transcription factor that recognizes TATA motif, and the RNA polymerase II complex to mediate basal transcription initiation [27]. On this basis, we inferred that the −228 to −3 bp region constitutes the core promoter of the *Rad21l* gene.

To further assess the promoter activity of these three regions, the serially truncated *Rad21l* promoter constructs were subjected to in vitro methylation, and their LUC/REN ratios were measured (Figure 1C). The LUC/REN ratio was 0.140–0.090 for the −2304 to −1974 bp fragment, 0.090–0.066 for the −1974 to −1294 bp fragment, and 0.063–0.212 for the −228 to −3 bp fragment. These results further support the involvement of these three regions in the transcriptional regulation of *Rad21l*.

Taken together, the core promoter of the *Rad21l* gene may reside within the proximal AT-rich region from −228 to −3 bp. The two distal upstream regions (−2304 to −1974 bp and −1974 to −1294 bp) may act cooperatively to modulate the activity of the proximal core promoter, thereby affecting the basal transcriptional output of the gene. It should be noted that the reporter assays in this section were performed in 293T cells, which lack the transcriptional environment of meiotic germ cells. The results therefore reflect the promoter’s in vitro activity, and its regulatory pattern in the testis in vivo requires further validation.

### 3.2. Association Between Upstream CpG Island Methylation and Tissue-Specific Expression of the Mouse Rad21l Gene

**Figure 2:**
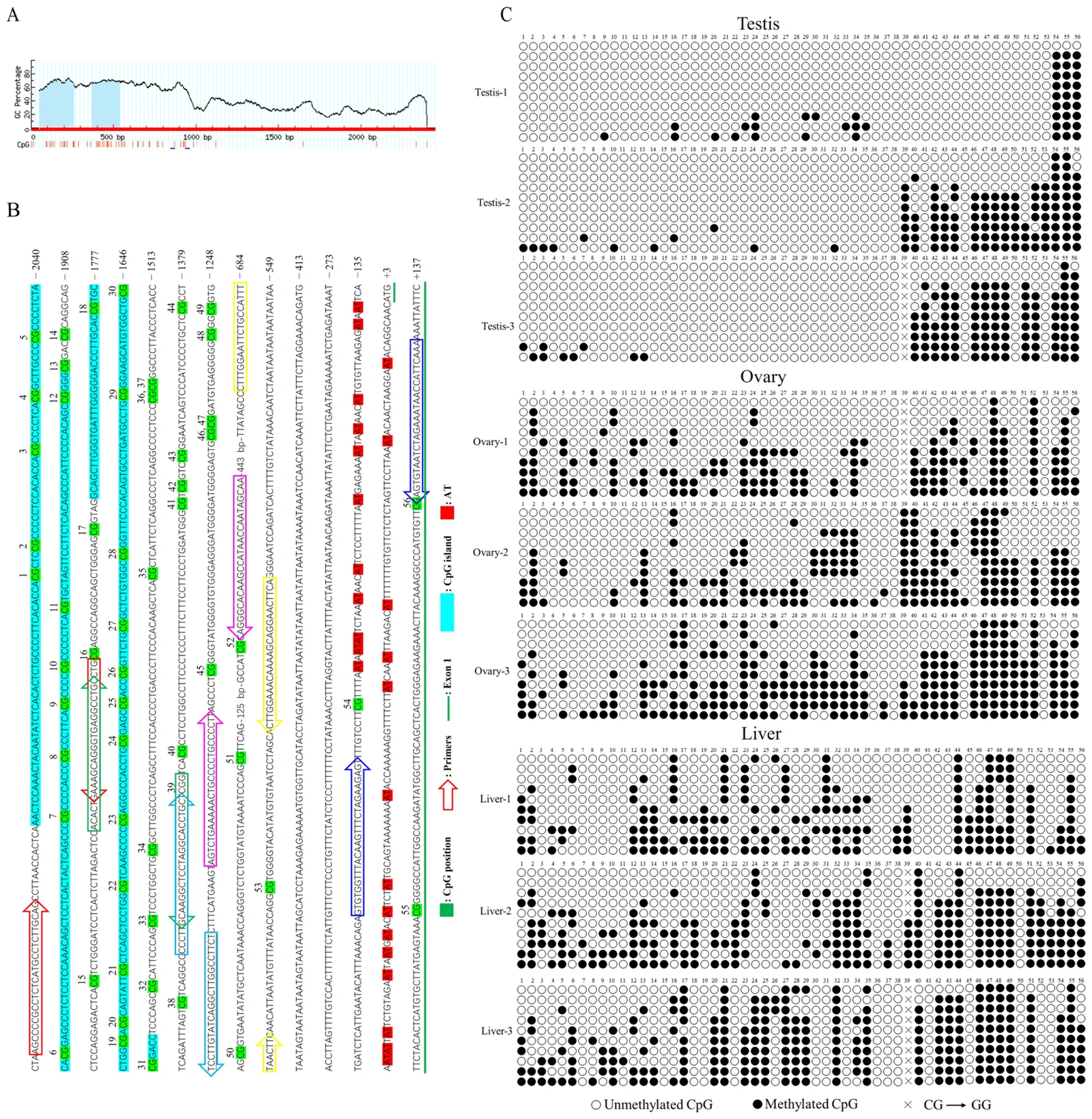
Prediction of CpG islands in the upstream regulatory region of *Rad21l* and analysis of tissue-specific DNA methylation. (**A**) Bioinformatic prediction of CpG islands (blue regions) within the sequence upstream of *Rad21l*. (**B**) Schematic of the *Rad21l* upstream regulatory fragment analyzed in this study. All 56 CpG dinucleotides within this region are indicated in green. (**C**) Bisulfite sequencing analysis of DNA methylation in this regulatory region in testis, ovary, and liver.

The serial truncation dual-luciferase assays and in vitro methylation experiments indicated that the two upstream distal regions, −2304 to −1974 bp and −1974 to −1294 bp, play important roles in regulating *Rad21l* transcriptional activity. DNA methylation modifications within these two regions may act on the proximal AT-rich region to achieve transcriptional regulation. Bioinformatic analysis identified CpG island 1 within the −2304 to −1974 bp region and CpG island 2 within the −1974 to −1294 bp region (Figure 2A,B). The *Rad21l* regulatory sequence examined in this study contained 56 CpG dinucleotide sites; CpG1–13 were located within CpG island 1, whereas CpG18–31 were distributed within CpG island 2 (Figure 2B). To investigate whether these two CpG-rich regulatory regions regulate the transcription of *Rad21l* through DNA methylation, we measured the methylation levels of all CpG sites, including those in the two CpG islands, in testis, ovary, and liver tissues (Figure 2C).

Methylation analysis showed that, in the testis, CpG1–38, CpG45, and CpG50 were unmethylated or hypomethylated; CpG40–44, CpG46–49, and CpG51–53 were partially methylated; and CpG54–56 were highly methylated. In the ovary, CpG3, CpG4, CpG6–9, CpG11, CpG12, CpG14, CpG15, CpG22, CpG35–38, CpG45, CpG51, and CpG53 were unmethylated or hypomethylated; CpG1, CpG2, CpG5, CpG10, CpG13, CpG16–21, CpG24–33, CpG40, CpG41, CpG43, CpG44, CpG46, CpG50, and CpG56 were partially methylated; and CpG23, CpG34, CpG42, CpG47–49, CpG52, CpG54, and CpG55 were highly methylated. In the liver, CpG7–9, CpG11, CpG15, CpG19, CpG22, CpG26, CpG30, CpG36–38, CpG41, CpG45, and CpG50 were unmethylated or hypomethylated; CpG1–6, CpG10, CpG12–14, CpG16–18, CpG20, CpG23–25, CpG27–29, CpG31, CpG33–35, CpG40, CpG42, CpG43, CpG52–54, and CpG56 were partially methylated; and CpG21, CpG32, CpG44, CpG46–49, CpG51, and CpG55 were highly methylated. Notably, methylation levels at CpG1–38 differed significantly among tissues. These sites were predominantly hypomethylated in the testis, with a mean methylation level of 3.246, whereas methylation levels were higher in the ovary and liver, with mean values of 27.018 and 33.860, respectively. Owing to its unstable methylation status and CG-to-GG base variation in some samples, the CpG39 site was excluded from statistical analyses.

We further analyzed the correlation between methylation at each site and *Rad21l* expression levels across tissues (Table 3). The methylation levels of multiple CpG sites were significantly correlated with *Rad21l* expression. In particular, methylation at 13 CpG sites—CpG1, CpG2, CpG5, CpG13, CpG16, CpG20, CpG21, CpG23, CpG25, CpG26, CpG28, CpG32, and CpG34—showed a significant negative correlation with *Rad21l* expression (Table 3).

Potential transcription factor-binding sites encompassing the 56 CpG sites were further predicted using the JASPAR database, and only a subset of the CpG sites was located within predicted transcription factor-binding regions (Table 4). Comparison of these predictions with the sites showing a negative methylation–expression correlation identified CpG5 as a key site and KLF4 as the corresponding transcription factor. Previous studies have shown that KLF4 contains functionally independent transcriptional activation and repression domains. Depending on the methylation status of the promoter region, KLF4 can activate transcription by interacting with E1A binding protein p300 (p300), a histone acetyltransferase coactivator, or mediate transcriptional repression by recruiting histone deacetylase 3 (HDAC3), a class I histone deacetylase that condenses chromatin [28,29].

Collectively, these findings suggest that differential methylation of CpG islands 1 and 2 may contribute to the tissue-specific expression of *Rad21l*. In the ovary and liver, elevated CpG methylation may reduce the transcriptionally permissive state of these regulatory regions and thereby suppress *Rad21l* transcription. In contrast, hypomethylation of the two CpG islands in the testis may favor transcription factor-mediated activation of *Rad21l*, resulting in markedly higher expression in testicular tissue. In vitro methylation with M.SssI methylates all CpG sites in the plasmid. Therefore, this experiment demonstrates only that plasmid-wide CpG methylation can suppress promoter activity; it cannot attribute the suppressive effect entirely to either CpG island 1 or CpG island 2. Further site-specific methylation experiments are needed to distinguish the contributions of the two CpG islands.

### 3.3. Structural Basis of KLF4 Functional Regulation Through CpG Methylation-Induced Conformational Remodeling

To further elucidate the potential mechanism by which KLF4 regulates the transcriptional activity of the *Rad21l* gene, we selected a 24-bp DNA sequence containing the CpG5 site and generated unmethylated and methylated versions of this sequence. Molecular docking was then used to model KLF4 complexes with the two DNA fragments and investigate how CpG methylation might induce KLF4 conformational remodeling and thereby regulate its transcriptional function.

**Figure 3:**
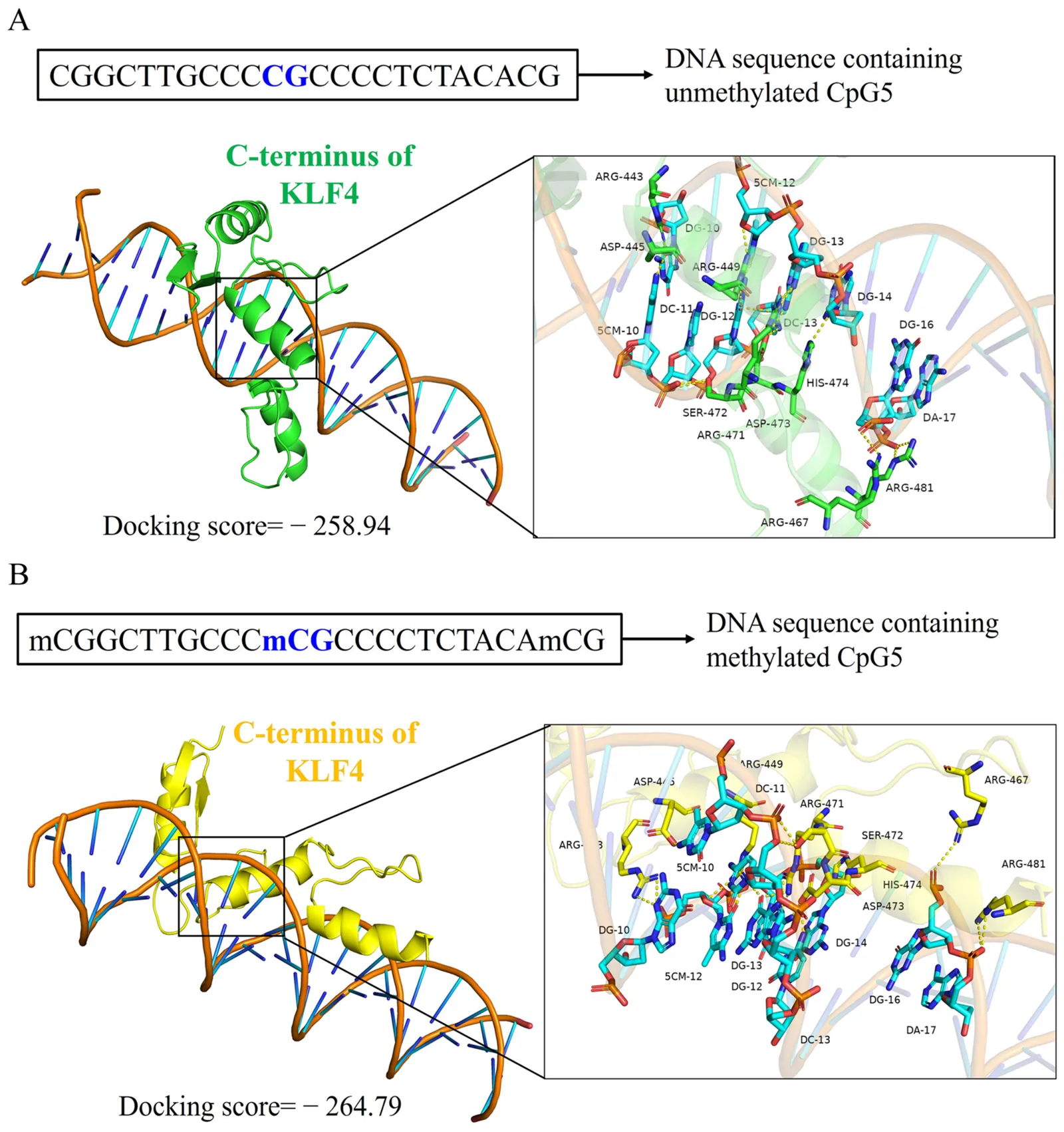
Comparison of molecular docking models of KLF4 with unmethylated and methylated DNA sequences containing CpG5. (**A**) Docking complex of KLF4 with the unmethylated DNA sequence containing CpG5 (blue sequence); the enlarged view shows local protein-DNA hydrogen-bonding interactions. (**B**) Docking complex of KLF4 with the methylated DNA sequence containing CpG5 (blue sequence); the enlarged view indicates the 5-methylcytosine modification site. Yellow dashed lines indicate hydrogen bonds between amino acid residues and nucleotides.

The docking simulations yielded a score of −258.94 for the unmethylated version and −264.79 for the CpG-methylated version. In both DNA substrate versions, the C-terminal zinc-finger domain of KLF4 inserted into the major groove of double-stranded DNA and formed a stable hydrogen-bond network with the core CG motif through conserved polar residues, including Arg449, Arg471, His474, and Arg481 (Figure 3). These simulations suggest that CpG methylation does not prevent KLF4 from binding the target promoter DNA and that KLF4 has the structural potential to recognize and anchor to methylated CG elements, in contrast to methylation-sensitive transcription factors such as SP1.

Although stable protein–DNA binding was achieved in both modeled systems, the global spatial conformations of the two complexes differed markedly. When bound to unmethylated promoter DNA, KLF4 adopted an extended overall conformation, with its N-terminal activation domain fully exposed to solvent (Figure 3A). This suggests that KLF4 may retain an intact interface for interaction with the p300 coactivator and have the structural potential to recruit p300, establish an open chromatin state, and activate target gene transcription.

When the sequence containing CG sites was methylated, the introduced 5-methylcytosine residues (5CM-10 and 5CM-12) altered the electrostatic and hydrophobic microenvironment within the DNA groove. The simulations suggest that this change could induce global conformational rearrangement of KLF4 (Figure 3B). This rearrangement may change the spatial orientation of the protein’s N-terminal region, causing the activation domain to fold inward and become occluded, disrupting the molecular interface required for p300 binding and eliminating its ability to recruit the coactivator complex. At the same time, conformational remodeling may expose the protein’s repression domain and create conditions for recruitment of the HDAC3 repressive complex, ultimately mediating repression of target gene transcription.

Together, these structural simulations support the hypothetical model proposed in this study: promoter methylation may act as a molecular switch that changes KLF4 conformation and cofactor selection, rather than displacing KLF4 from DNA, thereby mediating bidirectional regulation of *Rad21l* transcription. The proposed changes in p300 and HDAC3 recruitment are based solely on molecular docking predictions and require experimental validation.

### 3.4. Transcriptomic Characteristics and Functional Enrichment Associated with KLF4-Mediated Regulation of Rad21l

The preceding molecular docking and bioinformatic analyses suggested that KLF4 can specifically bind the CpG5-containing sequence of the *Rad21l* gene in both its methylated and unmethylated states, providing a structural basis for the association between KLF4 and the *Rad21l* gene. To further characterize the molecular network and biological functions associated with KLF4-mediated *Rad21l* regulation at the transcriptomic level, transcriptome sequencing was performed on representative testis, ovary, and liver samples.

**Figure 4:**
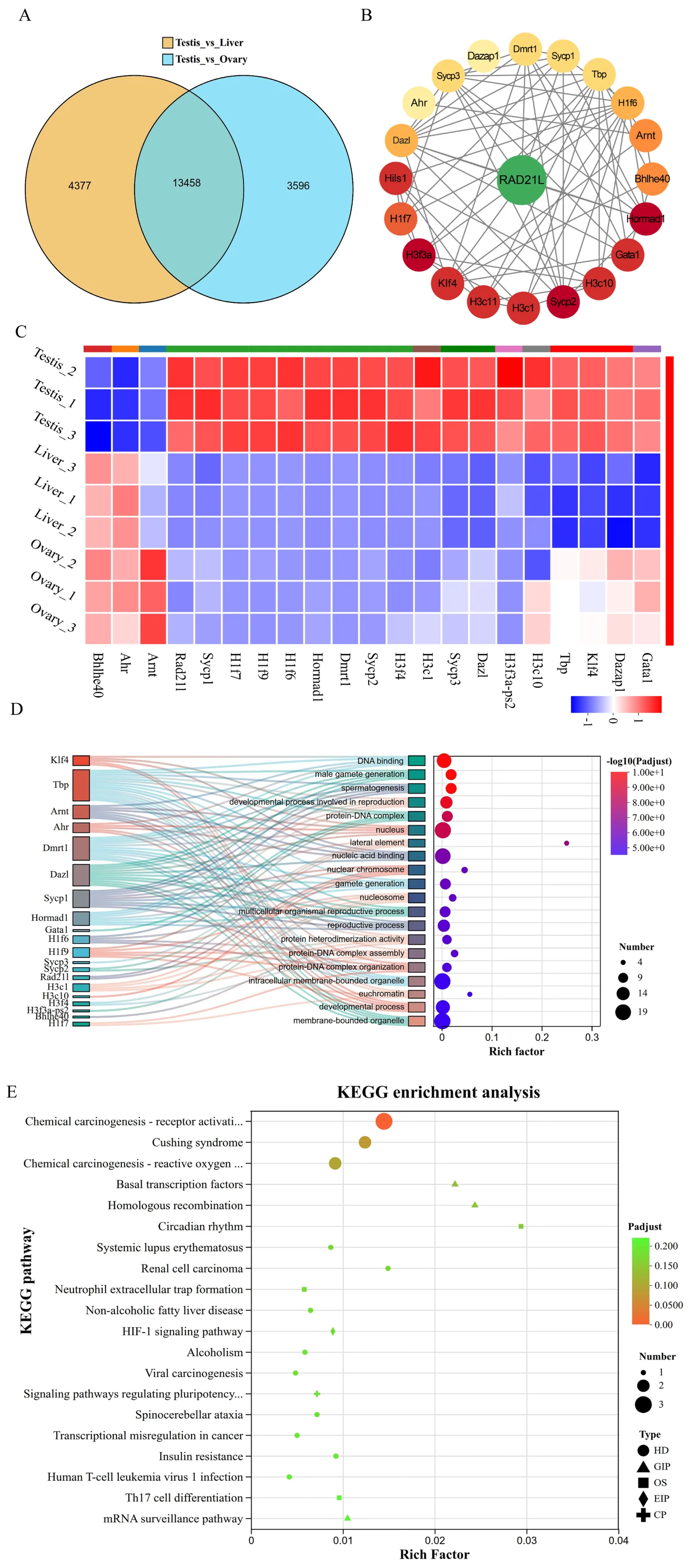
Transcriptomic profiles and functional enrichment analyses of three mouse tissues. (**A**) Venn diagram of differentially expressed genes among the tissues. (**B**) Protein–protein interaction network of 20 *Rad21l*-related genes selected from the set of differentially expressed genes. (**C**) Heatmap showing the expression of the 20 *Rad21l*-related genes in testis, ovary, and liver. From top to bottom, each set of three rows corresponds to testis, liver, and ovary, respectively; gene names are shown on the x-axis. Red and blue indicate high and low expression, respectively. (**D**) Sankey diagram and bubble plot of GO enrichment for the candidate gene set. (**E**) Bubble plot of KEGG pathway enrichment for the candidate gene set.

A total of 13,458 differentially expressed genes were identified (Figure 4A). Based on published evidence and the regulatory framework proposed in this study, 20 transcription factors and functional genes closely related to *Rad21l* function and transcriptional regulation were selected to generate a core gene set (Figure 4B), and their expression profiles were visualized using a heatmap (Figure 4C). This gene set included genes encoding canonical histones H1 and H3, providing candidate molecules for subsequent investigation of potential RAD21L-interacting partners.

The candidate genes displayed pronounced tissue-specific expression patterns. BHLH family member e40 (*Bhlhe40*), aryl hydrocarbon receptor nuclear translocator (*Arnt*), and aryl hydrocarbon receptor (*Ahr*) were preferentially expressed in the ovary. In contrast, *Rad21l*, members of the synaptonemal complex protein (*Sycp*) family (meiosis-related structural proteins), members of the hepatic leukemia factor (*Hlf*) family (transcriptional regulators), and genes encoding histones H1 and H3 generally showed low expression in the liver. GATA-binding protein 1 (*Gata1*, a zinc-finger transcription factor), DAZ-associated protein 1 (*Dazap1*, a synaptonemal complex component essential for spermatogenesis), and the core transcription factor *Klf4* were highly expressed in the testis. The testis-specific high expression of *Klf4* is consistent with the aforementioned hypothetical model in which KLF4 targets and regulates *Rad21l*, suggesting a potential molecular basis for KLF4-mediated transcriptional regulation of *Rad21l* in the testis. Histone H1- and H3-encoding genes also showed clear tissue-dependent differences in expression, suggesting a potential association with the tissue-specific chromatin environment relevant to RAD21L function.

To characterize the biological functions and regulatory properties of the candidate gene set, GO enrichment analysis was performed (Figure 4D). The selected genes were significantly enriched in molecular functions and biological processes related to DNA binding, nucleic acid binding, protein–DNA complex assembly, spermatogenesis, and male gamete generation. Sankey network analysis illustrated the relationships between individual genes and enriched functional categories. Notably, *Klf4* was associated with DNA-binding functions, consistent with its predicted ability to interact with the *Rad21l* regulatory sequence.

KEGG pathway enrichment analysis further showed that the candidate genes were mainly associated with pathways related to basal transcription factors, HIF-1 signaling, and signaling pathways regulating pluripotency of stem cells (Figure 4E). In particular, enrichment of transcription-related pathways was consistent with the GO analysis and indicated that the selected gene set is broadly involved in transcriptional regulatory networks.

Overall, together with the structural evidence supporting KLF4 binding to the CpG5-containing *Rad21l* sequence, the transcriptomic data provide complementary expression- and pathway-level support for a potential role of KLF4 in tissue-associated transcriptional regulation of *Rad21l*. These findings provide an omics-level framework for further investigating how CpG methylation may influence the transcriptional regulatory state of KLF4.

### 3.5. Identification and Structural Analysis of α-Kleisin Interactions with Histones and Chromatin Regulatory Proteins

**Figure 5:**
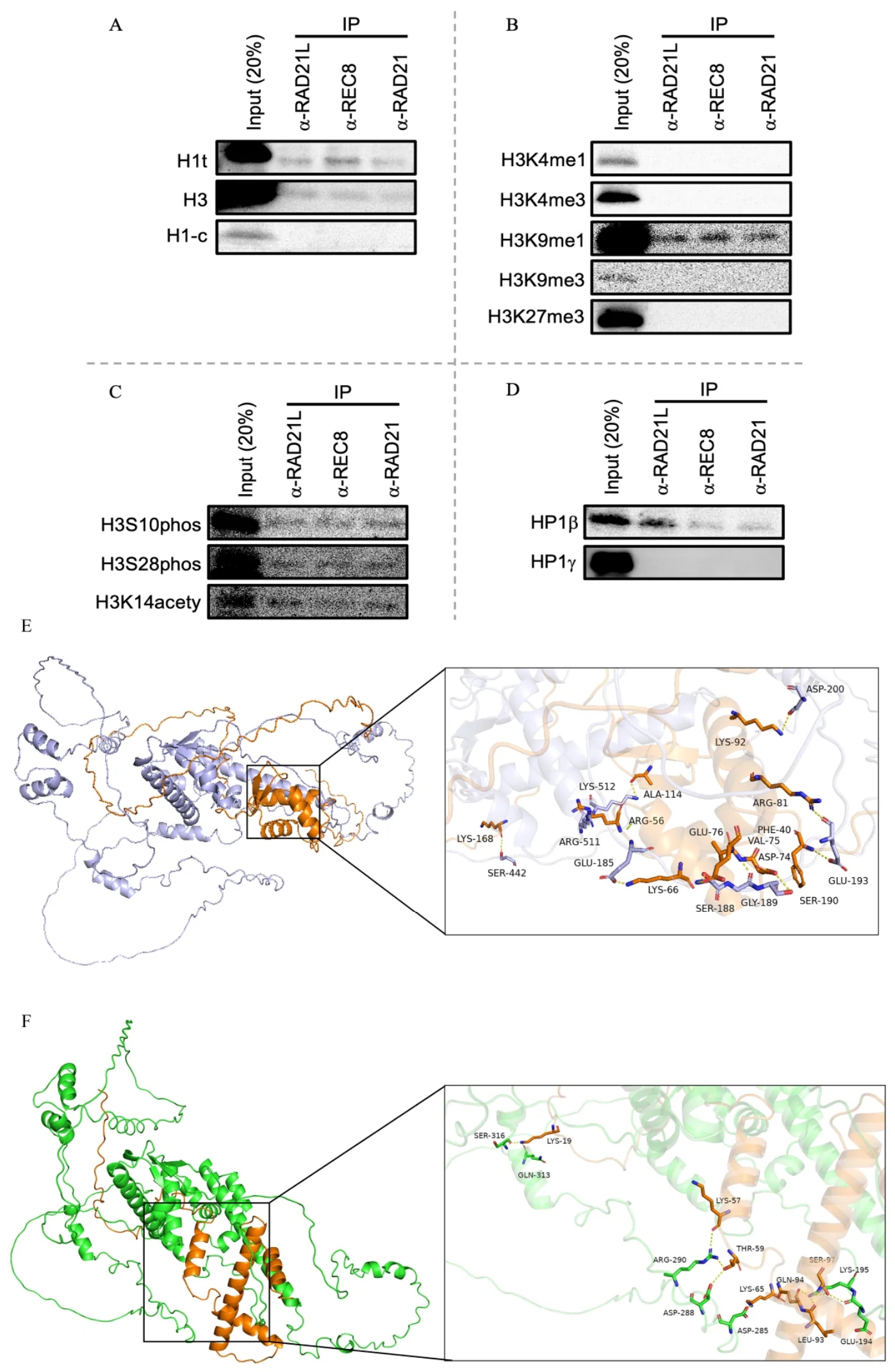
Analysis of α-kleisin associations with histones and chromatin-regulatory proteins and molecular docking models of RAD21L-histone binding. (**A**–**D**) Testis extracts were subjected to co-IP using antibodies against the indicated α-kleisin subunits. The immunoprecipitates and aliquots of the input fractions were analyzed by immunoblotting with the indicated antibodies. (**E**) Molecular docking model of RAD21L (blue-violet) with H1t (orange). The enlarged view on the right shows key amino acid residues at the binding interface; yellow dashed lines indicate hydrogen bonds between amino acid residues. (**F**) Molecular docking model of RAD21L (green) with H3 (orange). The enlarged view on the right shows key amino acid residues at the binding interface; yellow dashed lines indicate hydrogen bonds between amino acid residues.

Transcriptomic analysis suggested that histones H1 and H3 have the potential to interact with RAD21L. To investigate the interaction characteristics of α-kleisin complexes, we used mouse testicular protein extracts to examine the testis-specific histone H1t, histone H3, and multiple modified histone forms by co-IP. RAD21L, REC8, and RAD21 were each co-enriched with H1t and H3 in the same protein complexes, whereas no interaction signal was detected for the H1 C-terminal fragment examined in parallel (Figure 5A).

Given the stable co-occurrence of α-kleisin proteins with H3, we further screened several modifications of the H3 N-terminal tail. Among the histone methylation modifications examined, only H3K9me1 co-occurred with RAD21L, REC8, and RAD21 (Figure 5B). These α-kleisin proteins also co-occurred with H3 phosphorylated at serine 10 (H3S10phos, a serine phosphorylation mark involved in regulating mitotic and meiotic progression), H3 phosphorylated at serine 28 (H3S28phos, a serine phosphorylation modification important for regulating chromatin condensation and gene expression), and H3 acetylated at lysine 14 (H3K14ac, a lysine acetylation mark that mediates chromatin relaxation and is closely associated with transcriptional activation) (Figure 5C). These findings suggest that α-kleisin complexes preferentially co-enrich components bearing specific H3 N-terminal modifications in the co-IP assay. Co-IP establishes only the co-occurrence of proteins in a complex and cannot distinguish direct physical binding from indirectly mediated co-occurrence; therefore, these findings constitute only a preliminary screen of α-kleisin–histone interactions. The specific co-occurrence pattern of H3K9me1 may have a regulatory role in the chromatin localization of RAD21L complexes. In addition, RAD21L, REC8, and RAD21 were all co-enriched with the chromatin regulatory protein HP1β (Figure 5D).

To characterize the structural basis of these interactions, protein–protein docking simulations were performed between RAD21L and either H1t or H3. The docking models were constructed using three-dimensional histone structures lacking post-translational modifications, with the aim of characterizing the fundamental binding modes between RAD21L and the histone scaffold. Docking analysis of RAD21L and H1t suggested that the two proteins could form a stable complex. At the protein–protein interface, histone H1t (Figure 5E, orange) interacted with RAD21L (Figure 5E, blue-violet) through numerous hydrogen bonds and salt bridges. Basic residues of H1t, including Lys66, Arg56, Arg81, and Lys92, were predicted to form an extensive hydrogen-bonding network with polar and acidic residues of RAD21L, such as Glu185, Asp200, and Glu193. The hydrophobic H1t residues Phe40 and Val75 also contributed to the stability of the binding interface (Figure 5E). Similarly, histone H3 (Figure 5F, orange) interacted with RAD21L (Figure 5F, green) through multiple hydrogen bonds and salt bridges at the protein–protein interface. The basic H3 residues Lys19, Lys57, and Lys65 were predicted to form an extensive hydrogen-bonding network with the polar and acidic RAD21L residues Gln313, Ser316, Asp285, and Asp288. In addition, Thr59 and Gln94 of H3 participated in hydrogen-bond formation, whereas the hydrophobic residue Leu93 contributed to the stability of the binding interface (Figure 5F). In the static docking model, residues corresponding to H3 N-terminal methylation and phosphorylation sites were not observed to participate directly in interfacial hydrogen-bond formation.

This pattern is consistent with the intrinsic structural properties of histone tails: the H3 N-terminus is an intrinsically disordered, flexible region, and conventional static protein docking cannot fully capture its dynamic conformations or modification-mediated weak interactions [19]. Based on previously reported models of histone modification recognition, it can be hypothesized that RAD21L primarily forms stable binding interfaces with the globular domains of H1t and H3, providing a structural basis for protein complex formation. Methylation and phosphorylation of the H3 N-terminus may not directly contribute hydrogen bonds at these interfaces; instead, by altering local charge distribution and peptide-chain flexibility, they may regulate the binding of RAD21L to histones through long-range allosteric effects. This could ultimately produce distinct patterns of α-kleisin complex co-occurrence with different modified H3 forms [22]. This mode of action, in which modifications regulate interaction strength without direct binding to the modified sites, has been widely reported for chromatin-binding proteins [23].

Taken together, the co-IP screening results and molecular docking simulations preliminarily indicate that RAD21L and other α-kleisin proteins can form complexes with the testis-specific histone H1t and histone H3, and that the H3 N-terminal region is a key functional region for maintaining these complexes. RAD21L may interact with the structural cores of H1t and H3 through two distinct amino acid interfaces. RAD21L complexes may selectively co-occur with H3 forms bearing modification marks and cooperate with HP1β in regulating chromatin structure, thereby affecting downstream gene transcription.

## 4. Discussion

### 4.1. CpG Methylation-Mediated Tissue-Specific Transcriptional Regulation of the Rad21l Core Promoter

*Rad21l* encodes a mammalian meiosis-specific cohesin α-kleisin that is functionally distinct from, yet cooperates with, the canonical meiotic cohesin REC8 to regulate germ cell meiosis. RAD21L has been implicated in spermatogonial proliferation, meiotic prophase chromatin remodeling, chromosome-axis organization, and transcriptional regulation [1,7]. Recent studies further indicate that RAD21L is not merely a structural cohesin component but also an important regulator of three-dimensional genome organization and germline-specific transcriptional programs in male germ cells, making its tightly controlled spatiotemporal expression essential for normal spermatogenesis and male fertility [12]. Previous studies have shown that *Rad21l* displays highly restricted reproductive tissue-specific expression, with high expression in mouse germline tissues but little or no expression in somatic tissues [9]. However, the upstream epigenetic mechanisms responsible for this restricted expression pattern have remained incompletely understood. In the present study, RT-qPCR analysis confirmed the strong testis-enriched expression pattern of *Rad21l*, supporting the notion that its spatiotemporal transcription is subject to stringent epigenetic regulation.

Using serial promoter-deletion constructs in dual-luciferase reporter assays, we identified major regulatory regions within the *Rad21l* promoter, and in vitro CpG methylation markedly reduced promoter activity. The decrease in reporter activity after M.SssI treatment suggests that CpG methylation represses *Rad21l* promoter activity. However, M.SssI can methylate all CpG sites on the plasmid, so this in vitro reporter assay alone cannot determine whether CpG island 1 or CpG island 2 is the specific region responsible for the repressive effect. Future targeted experiments, such as site-directed mutagenesis of individual CpG islands, could further identify the core functional region involved in this regulation. Promoter DNA methylation is a classical epigenetic mechanism associated with tissue-specific gene silencing. Increased methylation can reduce local chromatin accessibility, alter transcription factor occupancy, or facilitate the recruitment of transcriptionally repressive complexes, thereby contributing to stable tissue-dependent gene expression patterns [13,14]. In light of our findings, we propose that sustained hypermethylation of the *Rad21l* regulatory region in somatic tissues may contribute to transcriptional silencing, whereas hypomethylation in the testis may establish a more permissive regulatory environment for transcription factor-mediated activation. This provides a plausible epigenetic basis for the germline-enriched expression of *Rad21l*.

Bioinformatics predictions and molecular docking simulations in this study suggested that KLF4 could specifically bind the core *Rad21l* promoter sequence containing the CpG5 site and could bind both methylated and unmethylated promoter fragments. The docking results showed that CpG methylation could induce global conformational remodeling of KLF4, thereby altering its transcriptional regulatory potential. Considered alongside the transcriptomic functional enrichment results, KLF4 and its downstream transcriptional regulatory network differed significantly between tissues with high and low *Rad21l* expression, suggesting that KLF4 may be a key candidate upstream regulator of tissue-specific *Rad21l* transcription. KLF4 is a context-dependent transcriptional regulator that can exert activating or repressive effects depending on the methylation status of its target site. site. This context dependence is consistent with our proposed model, in which promoter hypomethylation in the testis favors an activation-competent KLF4 conformation that supports high *Rad21l* transcription. By contrast, promoter hypermethylation in the ovary and liver may favor an alternative KLF4 conformation with reduced activating capacity, thereby repressing *Rad21l* expression.

Growing evidence indicates that meiotic cohesins have dual roles in chromosome architecture and transcriptional regulation and that their tightly controlled spatiotemporal expression is closely linked to epigenetic regulation [1]. Studies of the related meiotic α-kleisin REC8 have shown that tissue-specific methylation of CpG sites within its core promoter is strongly associated with germline-specific expression, with promoter hypomethylation favoring transcription and hypermethylation suppressing promoter activity [11]. The regulatory pattern observed for *Rad21l* in the present study is consistent with this broader framework and suggests that DNA methylation-dependent regulation may represent a shared feature of meiosis-specific α-kleisin genes. Recent work has also linked DNA methylation to RAD21L-associated germ cell reprogramming and spermatogenic processes [12], further highlighting the importance of epigenetic regulation in RAD21L biology.

Importantly, remodeling of three-dimensional genome organization does not necessarily depend solely on altered binding of architectural proteins such as CCCTC-binding factor (CTCF), a key insulator protein that shapes chromatin topology, and RAD21. Dynamic changes in histone modifications can themselves contribute to differential chromatin interactions, suggesting that epigenetic modifications may regulate both transcriptional activity and higher-order chromatin architecture through multiple interconnected mechanisms [30]. By integrating promoter CpG methylation, KLF4-dependent transcriptional regulation, and tissue-specific *Rad21l* expression, the present study extends the current model of upstream *Rad21l* regulation and supports promoter methylation as an important epigenetic mechanism controlling meiosis-specific α-kleisin gene expression.

However, our findings are based primarily on in vitro reporter assays and structural simulations. Further in vivo experiments, including ChIP-qPCR and KLF4 perturbation, are needed to verify differences among tissues in *Rad21l* promoter methylation levels and actual KLF4 binding, thereby strengthening the in vivo evidence for this epigenetic transcriptional regulatory mechanism.

### 4.2. Molecular Mechanism by Which α-Kleisin Proteins May Achieve Selective Localization to Meiotic Chromatin Through Histone-Specific Interactions

Precise recruitment of meiotic cohesin complexes to chromosomes is essential for homologous chromosome pairing, synapsis, recombination, and accurate segregation. Compared with REC8, which primarily mediates sister-chromatid cohesion, RAD21L has broader roles in higher-order meiotic chromatin remodeling, chromatin-loop formation, and local transcriptional regulation and therefore contributes to meiotic homeostasis in male germ cells [5,6]. Recent studies have further shown that RAD21L contributes to the remodeling of germ cell three-dimensional genome topology and the regulation of meiosis-related transcriptional programs [1]. However, the molecular basis by which RAD21L recognizes specialized meiotic chromatin environments and achieves selective chromosomal localization remains poorly understood. In addition, transcription itself can act as a mobile barrier to cohesin-mediated loop extrusion, enabling the formation of local topological boundaries independently of fixed CTCF boundaries and highlighting the close coupling between transcription and chromatin architecture [31].

Transcriptomic screening combined with co-IP experiments in this study suggested that α-kleisin family proteins can co-occur with the testis-specific linker histone H1t and the core histone H3 and form protein complexes with them. Further analysis based on the co-IP results and molecular docking simulations indicated that the flexible N-terminal tail of H3 is a key structural region for maintaining the association within these complexes. No interaction signal was detected for the H1 C-terminal fragment in this study. Co-IP can establish only protein co-enrichment and cannot distinguish direct physical binding from indirect interactions. Therefore, these results provide only a preliminary basis for screening and identifying α-kleisin–histone interactions. H1t is a characteristic germline-specific linker histone variant expressed during male meiosis and is highly enriched in pachytene spermatocytes. Compared with somatic H1 variants, H1t exhibits relatively weak nucleosome binding and has been associated with a more relaxed meiotic chromatin environment, which may facilitate chromatin remodeling, accessibility of chromatin-associated proteins, and transcription of meiotic genes [32]. The observed co-occurrence of RAD21L and H1t in protein complexes provides a preliminary explanation for RAD21L’s preferential localization to meiotic chromatin. It suggests that RAD21L is not loaded onto chromatin randomly but can achieve targeted anchoring to meiotic chromosomes by recognizing germline-specific histone variants.

Screening for associations with multiple post-translational modifications of the H3 N-terminal tail revealed a potential preference of α-kleisin family proteins for particular histone modifications: among the classical methylation marks examined, a co-occurrence signal was detected specifically with H3K9me1, and co-occurrence was also observed with activation-associated marks such as H3S10phos, H3S28phos, and H3K14ac. H3K9me1 is often enriched in transcriptionally active euchromatic regions, whereas no co-occurrence signal was detected for H3K9me3, a characteristic heterochromatin mark. This preliminarily suggests that RAD21L preferentially localizes to meiotic euchromatin, consistent with its proposed roles in chromatin remodeling and local transcriptional regulation [33]. RAD21L was also co-enriched with the chromatin regulatory protein HP1β, suggesting that it may cooperate with heterochromatin regulators to maintain the dynamic balance at euchromatin–heterochromatin boundaries and the structural stability of meiotic chromatin. Studies of centromeric chromatin similarly indicate that the balance between euchromatic and heterochromatic histone states is important for higher-order chromosome architecture and that histone modifications function not merely as static epigenetic marks but can actively contribute to the organization of three-dimensional chromatin interactions [34]. Furthermore, some histone-modifying enzymes can influence chromosome structure and transcription independently of their catalytic activities, suggesting that structural or scaffolding functions of chromatin-associated proteins should also be considered [35].

This study further used molecular docking to examine the spatial binding modes of RAD21L with H1t and H3. The simulations clearly indicated that multiple hydrogen bonds between amino acid residues could form a stable binding framework in both complexes. Notably, the static docking models did not identify direct contacts between the examined post-translationally modified residues and the predicted binding interface. This observation is consistent with the intrinsic structural properties of histone tails. The H3 N-terminal tail is highly flexible and intrinsically disordered, and conventional static structural modeling is generally limited in its ability to capture weak, dynamic, modification-dependent interactions and conformational ensembles [4,19].

Taken together with previous models of histone modification recognition, our findings support a possible mechanism in which RAD21L establishes a basal interaction with the histone structural framework, whereas post-translational modifications of the H3 N-terminal tail modulate interaction strength indirectly through changes in electrostatic properties, local peptide flexibility, or longer-range conformational effects [22,23]. Such a mechanism could contribute to the preferential association of RAD21L with particular histone modification states without requiring the modified residue itself to form a direct hydrogen bond with RAD21L. Recent studies have demonstrated that the selective binding of cohesins to histones generally relies on mechanisms of structural binding plus modification-dependent allosteric regulation for cohesin-histone interactions [4], providing a potential framework for interpreting the complementary co-IP and docking results obtained here.

Together, these findings led us to propose a potential molecular model for RAD21L localization to chromosomes. In this model, α-kleisin family proteins achieve initial chromatin anchoring by recognizing the meiosis-specific histone H1t. Specific modification marks on the H3 N-terminal tail (H3K9me1, phosphorylation, and acetylation) guide precise selection of chromosomal regions, while cooperation with HP1β remodels local chromatin structure. These processes ultimately enable specific localization to meiotic chromosomes and, in turn, regulate higher-order chromatin structure and germline-specific transcriptional programs. This model is inferred from complex co-enrichment and structural simulations; a complete body of evidence for direct interactions is still lacking. Future studies using site-directed mutagenesis, perturbation of histone modifications, and cellular localization analyses could test how histone modifications regulate RAD21L loading onto chromosomes and its meiotic functions, thereby strengthening the experimental support for this mechanism.

## 5. Conclusions

In summary, this study systematically examined the potential mechanisms underlying the transcriptional regulation of the meiotic α-kleisin gene *Rad21l* and the chromosomal localization of its protein product, RAD21L, from the perspectives of epigenetic regulation and protein–chromatin interactions. At the transcriptional level, tissue-dependent CpG methylation within the *Rad21l* regulatory region was associated with its tissue-specific expression pattern. Promoter hypomethylation in the testis may generate a transcriptionally permissive environment that supports KLF4-dependent activation, whereas increased methylation in somatic tissues may reduce the transcriptional activation potential of the promoter and contribute to gene silencing. These findings support DNA methylation as an important epigenetic mechanism contributing to the tissue-specific expression of meiosis-related α-kleisin genes.

At the chromatin-recruitment level, RAD21L associated with the meiosis-related histone variant H1t, specific modified states of the H3 N-terminal region, and HP1β. These observations support a model in which histone composition, histone modification states, and chromatin regulatory proteins collectively influence the preferential localization of RAD21L to meiotic chromatin. This mechanism provides a potential explanation for the chromatin localization of α-kleisin and offers a framework for further elucidating higher-order chromatin remodeling and germline-specific transcriptional programs during meiosis.

## Figures and Tables

**Table 1:** PCR primers used to construct the dual-luciferase reporter vectors.

Primer Name	Fragment Size	Primer Sequence
mpRAD21L-F (-2695)	3000 bp	TAGCTAGCGTAAGCCATCACCCCTGGC
mpRAD21L-F (-2304)	2609 bp	TAGCTAGCCGCCTCTCTAAGCCCCGC
mpRAD21L-F (-1974)	2279 bp	TAGCTAGCGAGGCCTGCCTGCGAGGCCA
mpRAD21L-F (-1294)	1599 bp	CCGCTAGCTTATTTTAATCTAGTACCAAAA
mpRAD21L-F (-654)	959 bp	TAGCTAGCGGCGTGGGGTACATATGTG
mpRAD21L-F (-552)	857 bp	CCGCTAGCATAATAATAGTAATAATAATAG
mpRAD21L-F (-418)	723 bp	GTGCTAGCCAGATGACCTTAGTTTTGTCC
mpRAD21L-F (-228)	533 bp	GTGCTAGCCTAGAAGAGTTTTGTCCTTCG
mpRAD21L-F (-108)	413 bp	CCGCTAGCCATTCTATTGCAGTAAAAAA
mpRAD21L-F (-70)	375 bp	CCGCTAGCTTATCAAATTTAAGACATTTT
mpRAD21L-F (-30)	335 bp	CCGCTAGCCCTTAAATACAACTAAGGAATAC
mpRAD21L-F (-3)	308 bp	GTGCTAGCAACATGTTCTACACTCATGTGC
mpRAD21L-R (305)		GGAAGCTTCAGCAAAGCTTCATTTAAATCTATATTGG

The underline represents restriction enzyme cutting sites of *Nhe*I and *Hind*III.

**Table 2:** Primers used for bisulfite sequencing PCR analysis.

Primer Name	Fragment Size	Primer Sequence
MeRad21l-F1	333 bp	5′-AGTTTTGTTTTTTATGTTTTTTGTAG-3′
MeRad21l-R1		5′-CAAACAAACCTCACCCTACTTTC-3′
MeRad21l-F2	404 bp	5′-ATATTGAAAGTAGGGTGAGGTTTGT-3′
MeRad21l-R2		5′-CCAAACAAATACCTAAAAACCTTAC-3′
MeRad21l-F3	138 bp	5′-TTTTTGTAAGGTTTTTAGGTATTTGT-3′
MeRad21l-R3		5′-AAAAAACCAAACCTAATACAAAAAA-3′
MeRad21l-F4	313 bp	5′-AGTTTGAAAAATTGTTTTTGTTTTT-3′
MeRad21l-R4		5′-TTACTATTAATTATAACTTATACCCTAC-3′
MeRad21l-F5	103 bp	5′-TTTTGGAATTTTGTTATTTTAATTT-3′
MeRad21l-R5		5′-TAAAATTCCTACTTTTATTTCCAAA-3′
MeRad21l-F6	372 bp	5′-GTGTGGTTTATAAGTTTTTAGAAGAGT-3′
MeRad21l-R6		5′-TTTAAATAATTATTTCTAAATTACACTC-3′

**Table 3:** Correlation between *Rad21l* promoter methylation and expression.

CpG Position	CpG1	CpG2	CpG5	CpG13	CpG16	CpG20	CpG21	CpG23	CpG25	CpG26	CpG28	CpG32	CpG34	CpG56
Pearson’s (r-values)	−0.909	−0.884	−0.802	−0.938	−0.881	−0.72	−0.72	−0.923	−0.687	−0.668	−0.71	−0.767	−0.887	0.731
*p*-value	0.001	0.002	0.009	0.001	0.002	0.029	0.029	0.001	0.041	0.049	0.032	0.016	0.001	0.025

Pearson’s r-values ≥ ±0.3 and *p*-values ≤ 0.05 are considered to indicate significant correlations.

**Table 4:** Transcription factors predicted to bind CpG-containing regions of the *Rad21l* promoter.

Position	Transcription Factor	Score	Relative Score	Strand	Predicted Sequence
CpG1	Ahr::Arnt	8.84	1.00	−	GCGTG
CpG3	Ahr::Arnt	8.84	1.00	−	GCGTG
	Klf4	12.88	0.96	−	GGGGCGTGGT
CpG5	Klf4	14.58	0.98	−	GGGGCGGGGC
CpG7	Klf4	14.58	0.98	−	GGGGCGGGGC
CpG8	Klf4	13.49	0.97	−	AGGGCGGGGT
CpG9	Ahr::Arnt	8.84	1.00	−	GCGTG
CpG10	SP1	14.64	1.00	+	CCCCGCCCCC
CpG11	Arnt	10.35	1.00	+	CACGTG
	ARNT::HIF1A	9.08	1.00	−	ACGTG
	Mlxip	11.80	1.00	+	CACGTG
	Mycn	10.53	1.00	+	CACGTG
	Mlxip	12.14	0.97	−	GCACGTGA
	Bhlhe40	13.92	0.97	+	CTCACGTGCTA
CpG15	ARNT::HIF1A	9.08	1.00	−	ACGTG
	Smad4	10.51	0.96	+	CGTCTGG
CpG22	Foxn1	8.99	0.98	−	TGACGC
	CREB1	10.36	0.96	−	TGACGCCA
CpG33	Foxn1	10.06	1.00	−	GGACGC
CpG35	Ahr::Arnt	8.84	1.00	−	GCGTG
CpG40	Ahr::Arnt	8.84	1.00	−	GCGTG
	Pax2	8.23	0.97	+	GGTCACGC
CpG52	Gata1	7.02	1.00	−	CGATGG
CpG53	Ahr::Arnt	8.84	1.00	+	GCGTG

## Data Availability

The original contributions presented in this study are included in the article. Further inquiries can be directed to the corresponding author.

## References

[1] Marín-Gual L., Vara C., Sainz-Urruela R., Cuartero Y., Álvarez-González L., Felipe-Medina N., Garcia F., Llano E., Marti-Renom M.A., Pendás A.M. (2025). Meiotic cohesin RAD21L shapes 3D genome structure and transcription in the male germline. Sci. Adv..

[2] Zhang X., Liu Y., Wang N. (2025). Dynamic changes in histone lysine lactylation during meiosis prophase I in mouse spermatogenesis. Proc. Natl. Acad. Sci. USA.

[3] Carioscia S.A., Biddanda A., Starostik M.R., Tang X., Hoffmann E.R., Demko Z.P., McCoy R.C. (2026). Common variation in meiosis genes shapes human recombination and aneuploidy. Nature.

[4] Coßmann J., Kos P.I., Varamogianni-Mamatsi V., Assenheimer D.S., Bischof T.A., Kuhn T., Vomhof T., Papantonis A., Giorgetti L., Gebhardt J.C.M. (2025). Increasingly efficient chromatin binding of cohesin and CTCF supports chromatin architecture formation during zebrafish embryogenesis. Nat. Commun..

[5] Duffy M.E., Ngaw M., Polsky S.E., Marzec A.E., Zhang S.S., Dzierzgowski O.R., Nannas N.J. (2024). Mechanisms, Machinery, and Dynamics of Chromosome Segregation in *Zea mays*. Genes.

[6] Rong M., Matsuda A., Hiraoka Y., Lee J. (2016). Meiotic cohesin subunits RAD21L and REC8 are positioned at distinct regions between lateral elements and transverse filaments in the synaptonemal complex of mouse spermatocytes. J. Reprod. Dev..

[7] Ishiguro K.I., Fujiwara Y. (2026). Chromosome Architecture Defined by the Meiosis-Specific Cohesin in Mammalian Germ Cells. Adv. Exp. Med. Biol..

[8] Taniuchi Y., Hiraide K., Su R., Ijuin K., Wei X., Horii T., Hatada I., Lee J. (2023). Analysis of absolute amounts of two meiotic cohesin subunits, RAD21L and REC8, in mouse spermatocytes. J. Reprod. Dev..

[9] Biswas U., Hempel K., Llano E., Pendas A., Jessberger R. (2016). Distinct Roles of Meiosis-Specific Cohesin Complexes in Mammalian Spermatogenesis. PLoS Genet..

[10] Rong M., Feng N., Li J., Dalai W. (2025). CpG site methylation regulates mouse Rec8 gene promoter activity. J. Reprod. Dev..

[11] Ishiguro K., Watanabe Y. (2016). The cohesin REC8 prevents illegitimate inter-sister synaptonemal complex assembly. EMBO Rep..

[12] He C., Cui Y., Chen W., Li C., He Z. (2025). RAD21L1 Is Sufficient and Effective for Reprogramming Human Sertoli Cells to Phenotypic Spermatogonial Stem Cells Through DNA Methylation and Essential for Male Fertility. Adv. Sci..

[13] Atlasi Y., Stunnenberg H.G. (2017). The interplay of epigenetic marks during stem cell differentiation and development. Nat. Rev. Genet..

[14] Luo C., Hajkova P., Ecker J.R. (2018). Dynamic DNA methylation: In the right place at the right time. Science.

[15] Murase Y., Yokogawa R., Yabuta Y., Nagano M., Katou Y., Mizuyama M., Kitamura A., Puangsricharoen P., Yamashiro C., Hu B. (2024). In vitro reconstitution of epigenetic reprogramming in the human germ line. Nature.

[16] Alavattam K.G., Esparza J.M., Hu M., Shimada R., Kohrs A.R., Abe H., Munakata Y., Otsuka K., Yoshimura S., Kitamura Y. (2024). ATF7IP2/MCAF2 directs H3K9 methylation and meiotic gene regulation in the male germline. Genes Dev..

[17] Fang F., Li Z., Zhang X., Huang Q., Lu S., Wang X. (2024). Divergent Roles of KLF4 During Primordial Germ Cell Fate Induction from Human Embryonic Stem Cells. Reprod. Sci..

[18] Liu Y., Olanrewaju Y.O., Zheng Y., Hashimoto H., Blumenthal R.M., Zhang X., Cheng X. (2014). Structural basis for Klf4 recognition of methylated DNA. Nucleic Acids Res..

[19] Bayarsaikhan B., Zsidó B.Z., Börzsei R., Hetényi C. (2024). Efficient Refinement of Complex Structures of Flexible Histone Peptides Using Post-Docking Molecular Dynamics Protocols. Int. J. Mol. Sci..

[20] Pradeepa M.M., Rao M.R. (2007). Chromatin remodeling during mammalian spermatogenesis: Role of testis specific histone variants and transition proteins. Soc. Reprod. Fertil. Suppl..

[21] Mahadevan I.A., Kumar S., Rao M.R.S. (2020). Linker histone variant H1t is closely associated with repressed repeat-element chromatin domains in pachytene spermatocytes. Epigenet. Chromatin.

[22] Sehrawat P., Shobhawat R., Kumar A. (2022). Catching Nucleosome by Its Decorated Tails Determines Its Functional States. Front. Genet..

[23] Taverna S.D., Li H., Ruthenburg A.J., Allis C.D., Patel D.J. (2007). How chromatin-binding modules interpret histone modifications: Lessons from professional pocket pickers. Nat. Struct. Mol. Biol..

[24] Sokolova V., Miratsky J., Svetlov V., Brenowitz M., Vant J., Lewis T.S., Dryden K., Lee G., Sarkar S., Nudler E. (2024). Structural mechanism of HP1 -dependent transcriptional repression and chromatin compaction. Structure.

[25] Wu S., Doherty R., Persaud M., Greer K., Patil O., Jang J.K., McKim K. (2026). An interaction between HP1 and the Chromosomal Passenger Complex Initiates Acentrosomal Spindle Assembly in *Drosophila oocytes*. bioRxiv.

[26] Lee J., Hirano T. (2011). RAD21L, a novel cohesin subunit implicated in linking homologous chromosomes in mammalian meiosis. J. Cell Biol..

[27] Sugihara F., Kasahara K., Kokubo T. (2011). Highly redundant function of multiple AT-rich sequences as core promoter elements in the TATA-less RPS5 promoter of *Saccharomyces cerevisiae*. Nucleic Acids Res..

[28] Evans P.M., Zhang W., Chen X., Yang J., Bhakat K.K., Liu C. (2007). Krüppel-like Factor 4 Is Acetylated by p300 and Regulates Gene Transcription via Modulation of Histone Acetylation. J. Biol. Chem..

[29] Hu S., Wan J., Su Y., Song Q., Zeng Y., Nguyen H.N., Shin J., Cox E., Rho H.S., Woodard C. (2013). DNA methylation presents distinct binding sites for human transcription factors. Elife.

[30] Nie Y., Wang M. (2024). Dynamic Changes in Histone Modifications Are Associated with Differential Chromatin Interactions. Genes.

[31] Banigan E.J., Tang W., van den Berg A.A., Stocsits R.R., Wutz G., Brandão H.B., Busslinger G.A., Peters J.M., Mirny L.A. (2023). Transcription shapes 3D chromatin organization by interacting with loop extrusion. Proc. Natl. Acad. Sci. USA.

[32] Hayakawa K., Tani R., Nishitani K., Tanaka S. (2020). Linker histone variant H1T functions as a chromatin de-condenser on genic regions. Biochem. Biophys. Res. Commun..

[33] Peters A.H., O’Carroll D., Scherthan H., Mechtler K., Sauer S., Schöfer C., Weipoltshammer K., Pagani M., Lachner M., Kohlmaier A. (2001). Loss of the Suv39h histone methyltransferases impairs mammalian heterochromatin and genome stability. Cell.

[34] Schalch T., Steiner F.A. (2017). Structure of centromere chromatin: From nucleosome to chromosomal architecture. Chromosoma.

[35] Remsburg C., Jaramillo-Lambert A. (2026). Histone methyltransferase MET-2 differentially regulates autosome and X-chromosome structure and transcription during meiosis in *C. elegans*. bioRxiv.

